# Flowerlike hybrid horseradish peroxidase nanobiocatalyst for the polymerization of guaiacol

**DOI:** 10.3906/kim-2005-32

**Published:** 2020-10-26

**Authors:** Ersen GÖKTÜRK

**Affiliations:** 1 Department of Chemistry, Faculty of Arts and Science, Hatay Mustafa Kemal University, Hatay Turkey

**Keywords:** Horseradish peroxidase, hybrid nanoflowers, polymerization, guaiacol

## Abstract

In this study, the catalytic activity and stability of flowerlike hybrid horseradish peroxidase (HRP) nanobiocatalyst (HRP-Cu ^2+^ ) obtained from Cu ^2+^ ions and HRP enzyme in the polymerization reaction of guaiacol were analyzed. We demonstrated that HRP-Cu ^2+^ and hydrogen peroxide (H _2_ O _2_ ) initiator showed significantly increased catalytic activity and stability on the polymerization of guaiacol compared to that of free HRP enzyme. Poly(guaiacol) was observed with quite high yields (88%) and molecular weights (38,000 g/mol) under pH 7.4 phosphate-buffered saline (PBS) conditions at 60 °C with 5 weight% of HRP-Cu ^2+^ loading. HRP-Cu ^2+^ also shows very high thermal stability and works even at 70 °C reaction temperature; free HRP enzyme denatures at that temperature. Furthermore, HRP-Cu ^2+^ provided considerable repeated use and showed some degree of catalytic activity, even after the fourth recycle, in the polymerization of guaiacol.

## 1. Introduction

Enzyme biocatalysts are used in many scientific and technological fields such as chemistry, biochemistry, and medicine because of their high catalytic activity, selectivity, low toxicity, and water-soluble properties [1–7]. A variety of reactions can be catalyzed by enzymes under milder conditions and enzymes indicate activity against certain substrates or functional groups. For example, peroxidase enzymes achieve oxidation reactions of proton donor compounds with H _2_ O _2_ oxidizer/initiator. Peroxidase enzymes, such as horseradish peroxidase (HRP), contain iron (Fe) ions in their active centers [8]. These enzymes are used for radicalic reactions/polymerizations. Aniline and phenol derivatives can be successfully polymerized in the presence of HRP enzyme and H _2_ O _2_ . A variety of environmentally friendly polyaromatic structures can be synthesized in high yields. In addition to that, peroxidase enzymes can also oxidize different electron donor compounds including indoles, phenolic acids, and sulfates [9–16].

Peroxidase enzymes generally lose their activities at high reaction temperatures, acidic or basic conditions, and in organic solvents [6,17]. In addition, enzymes cannot be easily isolated from the reaction mixture, and therefore it is not easy to recycle enzymes for further reactions. These problems limit the use of enzymes in larger scale applications. To overcome these restrictions, immobilization of enzymes on solid support has been developed. The principal attribute of the immobilization method is to bind an enzyme on a support and limit its movement [18]. Immobilization of enzymes provides increasing reusability and more stable and economical catalyst systems. However, catalytic activities of many enzymes were lost after immobilization due to improper conformation and mass transfer restrictions between enzyme and substrate [19,20]. In order to use enzymes efficiently, discovering a suitable immobilization support exhibiting higher catalytic activities is very important in both commercial and scientific areas.

In recent decades, the nanoflower shaped nanobiomolecular catalysts, including immobilized enzymes, have been discovered to increase catalytic activity and stability [21–24]. The reasons behind the increased catalytic activity and stability of these nanoflower shaped immobilized enzymes are explained as 1) high surface area of nanoflowers, 2) less mass-transfer restrictions, 3) proper conformation of the enzyme in nanoflowers [21,22]. Hybrid nanoflower shaped biocatalysts were first discovered by Zare and coworkers [25]. They reported that the hybrid nanoflowers were produced from protein (bovine serum albumin) and Cu ^2+^ ions. Hybrid nanoflowers obtained from the immobilization of enzymes on metal ions exhibited higher catalytic activities and stability than the free enzymes [21]. Ocsoy and coworkers reported that the catalytic activity of the hybrid nanobiocatalyst formed by the HRP enzyme and Cu ^2+^ ions was quite high on the oxidation of guaiacol compared to using free HRP enzyme [26,27]. In addition, the aforementioned nanobiocatalyst was reported to show very high stability and reusability in contrast to free HRP enzyme. In these studies, only catalytic activity and stability of hybrid nanoflowers were examined, and no information related to the polymerization behavior of guaiacol or oxidation product was given. Guaiacol is a phenol derivative obtained from lignin. Guaiacol is used for the production of vanillin and as a building block for the synthesis of a variety of molecules [28]. Therefore, we believe that it is essential to show the polymerization tendency of guaiacol by using HRP-Cu ^2+^ hybrid nanoflowers.

## 2. Materials and methods

### 2.1. Materials

Methanol (Isolab, catalog# 947046), guaiacol (Sigma-Aldrich, catalog# W253200-1KG-K), pH 7.4 phosphate-buffered saline (PBS, MP biomedicals, catalog# 2810305), hydrogen peroxide (Merck, catalog# 1.08597), and copper (II) sulfate (CuSO _4_ , Sigma-Aldrich, catalog#18304) were used. Horseradish peroxidase (HRP) enzyme was purchased from Sigma-Aldrich (catalog#77332, lyophilized, powder, beige, ~150 U/mg) and used as received.

### 2.2. Characterization

^1^ and ^13^ C NMR analyses were carried out with a Bruker-Instruments-NMR DPX-400 spectrometer using the DMSO- *d6* solvent. Thermogravimetric analysis was carried out with a Mettler-Toledo TGA/DSC 1 Star system instrument in nitrogen atmosphere at the temperature changing from room temperature to 900 °C at a heating rate of 10 °C/min. The FT-IR spectra were performed on a Shimadzu IRAffinity-1S spectrometer. A Shimadzu LC-20AD instrument with an internal differential refractive index detector was used for gel permeation chromatography (GPC) analyses. Agilent PLgel mixed-B column was used with HPLC grade *N,N`* -dimethylformamide (DMF) mobile phase at 1 mL/min flow rate. Polydispersity polystyrene (PS) standards were used for calibration.

### 2.3. Preparation of HRP-Cu ^2+^ hybrid nanoflowers

Firstly, 120 mM CuSO _4_ solution was prepared in ultrapure water. CuSO _4_ (60 µL) was blended with 9 mL of free HRP enzyme solution (0.2 mg/mL concentration) in pH 7.4 PBS buffer. The obtained mixture was stirred for 5 min to allow homogenization and was left to incubate for three days at +4 °C. After centrifugation, the blue precipitate was washed with water to remove inert waste. The obtained nanoflowers were then dried and used for polymerization experiments [27].

### 2.4. Representative polymerization procedure

Guaiacol and HRP-Cu ^2+^ were mixed in 5 mL of a buffer solution. After that, the temperature of the mixture was adjusted to the desired reaction temperature. The compound H _2_ O _2_ (70 µL, 34.5%–36.5%) was added to the resulting solution 15 times every 10 min to initiate the polymerization. At the end of the reaction, the precipitated polymer was centrifuged. The obtained product was washed with water and methanol and dried at 60 °C [29,30]. The colors of the obtained products were black.

Poly(guaiacol) formation (Table 1; entry 4) = ^1^ NMR (400 MHz, DMSO- *d*_6_ ) δ ppm 2.50 (s,3H), 6.74 (m, 2H), 6.90 (dd, ^1^ ), 8.90 (s, ^1^ ). ^13^ C NMR (100 MHz, DMSO- *d*_6_ ) δ ppm 55.9, 112.7, 115.9, 119.6, 121.3, 146.9, 148.1.

**Table 1:** Polymerization of guaiacol by HRP-Cu ^2+^ in the presence of H _2_ O _2_ .

Entrya	HRP-Cu ^2+^ amount (mg)	pH	T _p_ (ºC)	Yield (%)	T _90_ (ºC)	T _50_ (ºC)	M _n_ (g/mol)	Ð
1	5	7.4	30	38	231	766	19000	1.05
2	5	7.4	40	55	239	776	23000	1.40
3	5	7.4	50	67	240	824	34000	1.14
4	5	7.4	60	88	252	828	38000	1.09
5	5	7.4	70	47	231	655	34000	1.17
6	10	7.4	60	90	245	809	35000	1.14
7	15	7.4	60	92	238	799	34000	1.20
8	5	8.0	60	68	235	683	13000	1.03
9	5	7.0	60	55	226	678	19000	2.42
10b	5	7.4	60	31	224	576	9700	1.11
11c	5	7.4	60	13	184	508	6000	1.09

^a^ All polymerizations were carried out with 100 mg of guaiacol in pH buffers in the presence of H _2_ O _2_ . ^b^ The reaction is conducted with a mixture of 4.5 mL of pH 7.4 PBS buffer and 0.5 mL of methanol. ^c^ The reaction is conducted with a mixture of 4.0 mL of pH 7.4 PBS buffer and 1.0 mL of methanol. T _p_ : the reaction temperature, T _90_ and T _50_ : the temperatures at 90% and 50% residues, respectively. M _n_ : the number average molecular weight, Ð: heterogeneity index.

### 2.5. Reusability experiments for the polymerization of guaiacol

The reusability of HRP-Cu ^2+^ was determined by consecutive polymerization reactions of guaiacol. After each reaction, 0.1 mL of HCl (0.1 M) was added to the mixture. The polymer product was centrifuged and separated from the reaction. Then, another run was started to monitor the next polymerization [31].

## 3. Results and discussion

**Figure 1:**
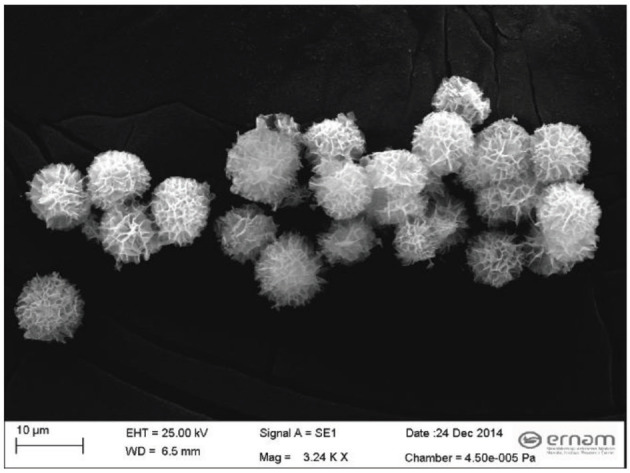
SEM image of synthesized HRP-Cu ^2+^ hybrid nanoflowers.

The coordination between Cu ^2+^ ions and amide nitrogen atoms in the protein structure of HRP enzyme formed complexes is the principal step for the formation of hybrid nanoflowers (HRP-Cu ^2+^ ) [27]. The Figure 1 demonstrates the morphology of the obtained nanoflowers using a scanning electron microscope (SEM). According to the SEM image of the nanoflowers, the obtained particles are micrometer sized, but they have nanoscale characteristics [25,27]. Therefore, the obtained catalyst was named a nanoflower. The formation of hybrid nanoflowers (HRP-Cu ^2+^ ) from copper ions (Cu ^2+^ ) and horseradish peroxidase (HRP) enzyme increases the catalytic activity and stability due to 1) high surface areas of the obtained nanoflowers, 2) favorable HRP conformation in HRP-Cu ^2+^ , and 3) entrapped HRP. Entrapped horseradish peroxidase enzyme with Cu ^2+^ ions can have more available active sites of HRP in the nanoflowers, and therefore, HRP-Cu ^2+^ shows higher catalytic activity and stability in contrast to free HRP enzyme. Previously, it was reported that the activity of HRP-Cu ^2+^ in the oxidation of guaiacol was found to be about 300% higher compared to the activity of the free HRP enzyme [27]. Inspired by this work, we discovered that the potential utilization of HRP-Cu ^2+^ for the polymerization of guaiacol means high efficiency, stability, and reusability under mild reaction conditions.

**Figure 2:**
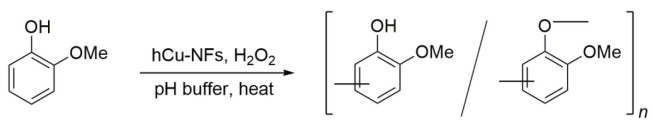
Polymerization of guaiacol by HRP-Cu ^2+^ hybrid nanoflowers and H _2_ O _2_ (hCu-NFs= HRP-Cu ^2+^ ).

The variable parameters including reaction pH, temperature, and the amount of HRP-Cu ^2+^ were optimized for the polymerization of guaiacol (Figure 2). All polymerizations were conducted in pH buffer solutions with the careful optimization of the amount of the catalyst and guaiacol to obtain the best polymerization conditions. To address the impact of the pH of the solution on the polymerization of guaiacol, we investigated three pH buffers: pH 7.0, 7.4, and 8.0 (Table 1). The results have shown that the optimum conditions for the polymerization of guaiacol were achieved in pH 7.4 phosphate-buffered saline (PBS). Polymerization attempts using pH 7.0 and 8.0 buffers (Table 1; entries 8 and 9) were also successful, however, obtained yields for those polymerizations were lower than the ones for the polymerization carried out in pH 7.4 PBS buffer.

The impact of reaction temperatures and the amount of HRP-Cu ^2+^ on the polymerization of guaiacol in pH 7.4 buffer were also investigated. When the polymerization was carried out with 5 weight% of catalyst loading at 60 °C in the presence of H _2_ O _2_ , the highest yield product (88%) was observed (Table 1; entry 4). Horseradish peroxidase is known to be thermally deactivated at temperatures around 60 °C due to the denaturation of the enzyme [32–34]. However, the highest yielded polymerization of guaiacol with HRP-Cu ^2+^ was achieved at 60 °C. According to the findings, HRP-Cu ^2+^ hybrid nanoflowers still work even at 70 °C reaction temperature (Table 1; entry 5). This achievement provides using HRP-Cu ^2+^ for oxidative polymerization reactions carried out at higher reaction temperatures. The optimum polymerization of guaiacol with 88% yield and 38,000 g/mol molecular weight was accomplished in pH 7.4 buffer at 60 °C with 5 weight% of HRP-Cu ^2+^ loading (Table 1; entry 4). Increasing the amount of HRP-Cu ^2+^ slightly increased the yield of the product (Table 1;entries 6 and 7), but the molecular weights of the obtained polymers were almost the same as the ones obtained under the conditions given in entry 4 in Table 1. Due to the high cost of HRP enzyme, the optimum HRP-Cu ^2+^ concentration was determined to be 5% by weight of guaiacol. The effect of the addition of an organic solvent to the reaction medium on the polymerization yield and molecular weight of the obtained product was also investigated (Table 1; entries 10 and 11). The stability of HRP-Cu ^2+^ was aimed to be determined by adding some amount of water-miscible organic solvents to the reaction media, because HRP enzyme is known to denature in organic solvents [15]. Polymerization performed using a mixture of 4.5 mL of pH 7.4 PBS buffer and 0.5 mL of methanol (Table 1; entry 10) under 60 °C reaction temperature with 5 weight % of catalyst loading showed a dramatic decrease in the yield (31%) and molecular weight (9700 g/mol) of the obtained polymer. Further increase in the amount of methanol to 1.0 mL with 4.0 mL of pH 7.4 PBS buffer under the same reaction conditions (Table 1; entry 11) resulted in losing a considerable degree of the catalytic activity of HRP-Cu ^2+^ and gave a polymer with 13% yield and 6000 g/mol molecular weight (see Supplementary information for GPC data).

**Figure 3:**
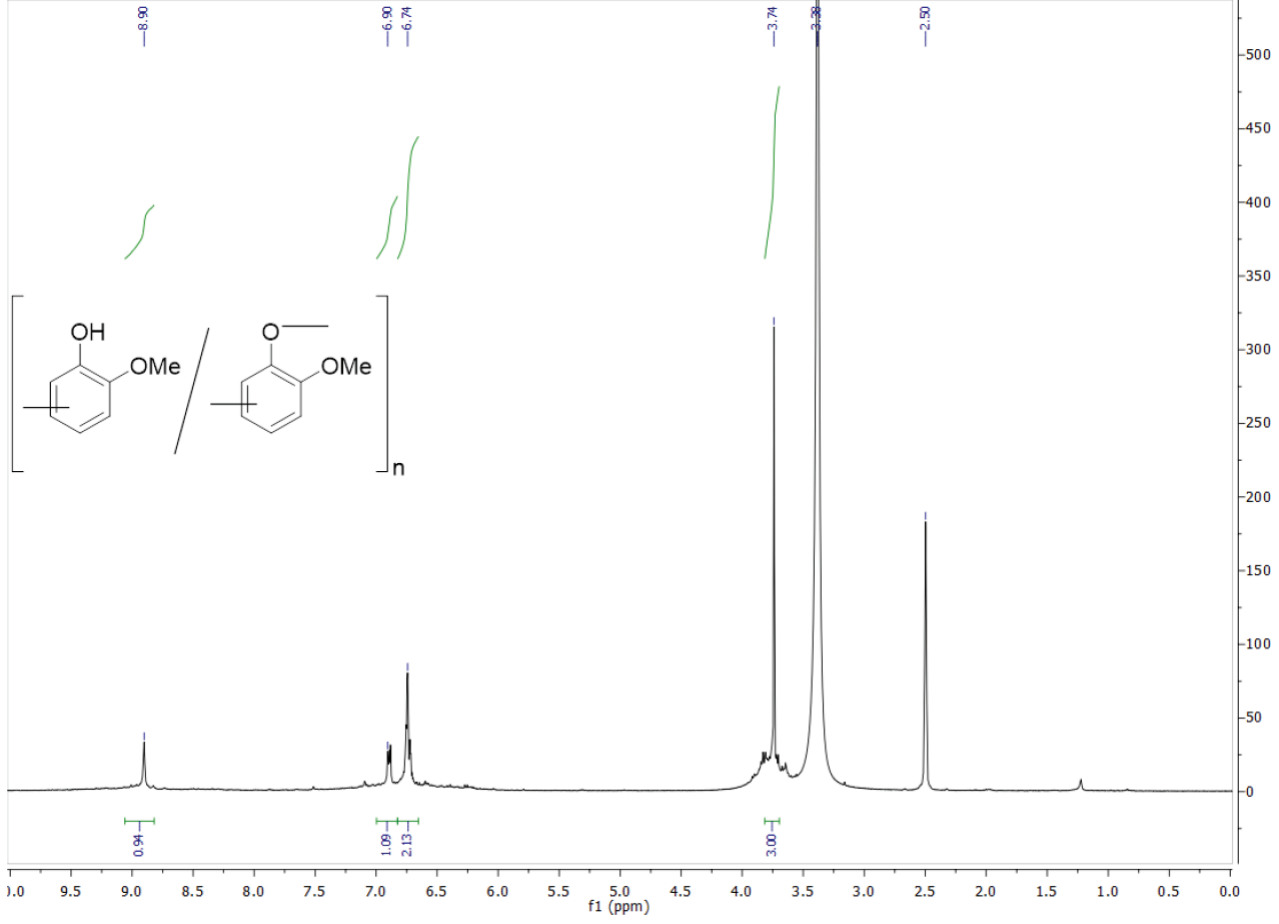
^1^ NMR spectrum of the product (Table 1; entry 4).

The structure of the resultant polymer (Table 1; entry 4) was studied by FT-IR, ^1^ , and13 C NMR spectra. To understand the structure of the resulting product, ^1^ NMR analysis was first investigated for polymer entry 4 in Table 1 (Figure 3). According to ^1^ NMR spectrum of the obtained product, methyl proton signals of the methoxy group in the polymer structure appeared as a singlet peak at 3.74 ppm. The expected chemical shifts for the aromatic protons of the product were observed at 6.74, 6.90, and 8.90 ppm, which confirmed the structure of poly(guaiacol). The proton of the phenolic –OH was not detected in the ^1^ NMR analysis of the product, suggesting that –OH protons of the product probably interacted with DMSO- *d6* to exchange hydrogen and deuterium atoms [35]. Therefore, –OH protons of the product disappeared in the ^1^ NMR spectrum of the product.

**Figure 4:**
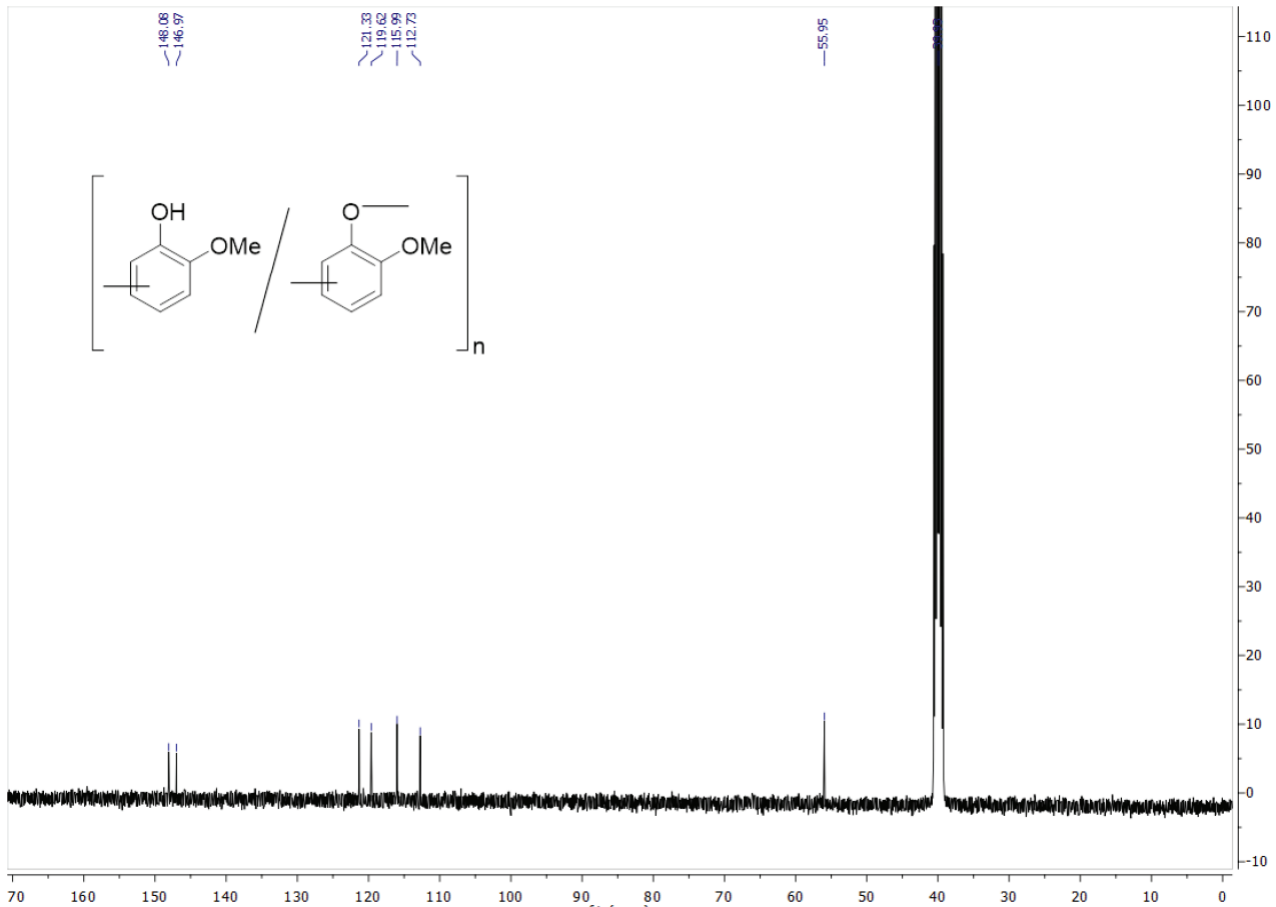
^13^ C NMR spectrum of obtained poly(guaiacol) (Table 1; entry 4).

Figure 4 displays the ^13^ C NMR spectrum for the obtained poly(guaiacol) (Table 1; entry 4). The presence of the methyl carbon of methoxy group can be recognized at δ= 56 ppm. The peaks observed between 112.7–148.1 ppm can be assigned as aromatic carbons of obtained poly(guaiacol). The ^13^ C NMR spectrum confirmed that the obtained product had a perfect agreement with the expected poly(guaiacol) structure.

**Figure 5:**
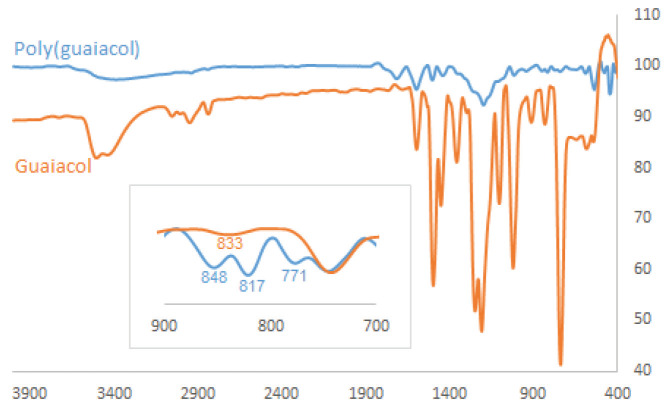
FT-IR spectra results of guaiacol and poly(guaiacol) (Table 1; entry 4).

Following these results, we evaluated the FT-IR analysis of guaiacol and poly(guaiacol) (Table 1; entry 4) to confirm the occurrence of polymerization (Figure 5). An absorption band that appeared around 1650 cm–1 in the FT-IR spectrum of the product was probably related to the formation of a benzoquinone type structure in the backbone. –OH stretching vibrations of guaiacol and the product were clearly matching around 3400 cm–1. The absorption bands at 817 and 848 cm–1 in the FT-IR spectrum of the product were attributed to 1,2,4-trisubstituted benzene ring formation in the polymer structure [36]. These peaks verified that polymerization was propagated through *ortho* - *ortho* or *ortho* - *para* couplings of guaiacol and verifying the formation of phenylene/oxyphenylene repeat units.

Thermogravimetric analysis was also performed to analyze the thermal stabilities of the obtained products. According to the literature, 10% weight losses of poly(guaiacol) synthesized from manganese based catalysts were reported to be between 100–200 °C, and 41% of initial weights of those products were found to remain after pyrolysis under nitrogen atmosphere [36,37]. According to TGA thermograms, the obtained polymers synthesized from an HRP-Cu ^2+^ hybrid catalyst have shown considerably higher thermal stabilities under nitrogen atmosphere (see Supplementary information for TGA data). The polymer obtained from Table 1, entry 4 conditions started to decompose around 200 °C and lost 10% and 50% of its initial weight at 252 °C and 828 °C, respectively. Pyrolysis residue (carbonaceous char) of the poly(guaiacol) (Table 1; entry 4) was found to be 48% at 900 °C. Since poly(guaiacol) has a long conjugated polyaromatic backbone, the obtained products demonstrated very high thermal stabilities.

Polymerization of guaiacol was also performed using free HRP enzyme in order to detect differences between activities of free HRP enzyme and HRP-Cu ^2+^ . The polymerization results of guaiacol by free HRP enzyme in the presence of H _2_ O _2_ are summarized in Table 2. The optimum polymerization of guaiacol with 51% yield and 9600 g/mol molecular weight was accomplished with 5 weight% of HRP enzyme in pH 7.4 PBS buffer at 30 °C (Table 2; entry 13). Increasing the reaction temperature to 40 °C (Table 2; entry 14) resulted in a decrease of the polymerization yield (35%) since HRP is sensitive to heating and it denatures around 60 °C [32–34]. Enhancing HRP concentration to 10 weight% (Table 2; entry 15) slightly increased the yield (53%) and molecular weight (10,800 g/mol) of the product. However, the addition of 15 weight% of HRP (Table 2; entry 16) resulted in a small increase of the yield (56%) and a decrease of the molecular weight (8400 g/mol) of the product compared to the conditions of entry 15 in Table 2. Since HRP is a very expensive enzyme, 5 weight% of HRP was decided to be in an optimum concentration in the polymerization of guaiacol (Table 2; entry 13). According to the obtained results, HRP-Cu ^2+^ was decided to have higher catalytic activity and stability in the polymerization of guaiacol compared to free HRP enzyme. Polymerization of guaiacol carried out with HRP-Cu ^2+^ gave poly(guaiacol) with higher yield and molecular weight compared to the free HRP enzyme. HRP-Cu ^2+^ catalyst exhibited higher stability at reaction temperatures of 60 °C and above, and it did not undergo denaturation in contrast to free HRP enzyme.

To examine the reusability of HRP-Cu ^2+^ , consecutive polymerizations of guaiacol were performed under pH 7.4 PBS buffer with 5 weight% HRP-Cu ^2+^ loading at 60 °C (Table 3). After each polymerization, the precipitated product was centrifuged and separated from the reaction media. After that, another run was started up with the addition of guaiacol to the reaction media to see polymerization yield and molecular weight. The second run (Table 3; entry 17) showed that the activity of HRP-Cu ^2+^ was similar to the first run (Table 1; entry 4). Polymerization yield was 54% and the number average molecular weight ( *M* n) of the obtained product was 14,000 g/mol. HRP-Cu ^2+^ still showed some activity in the third run, and gave poly(guaiacol) with 21% yield and 6000 g/mol molecular weight (Table 3; entry 18). The HRP-Cu ^2+^ hybrid nanoflowers showed slight catalytic activity even in the fourth run and gave a polymer with 8% yield with 2900 g/mol molecular weight (Table 3; entry 19). However, no product was observed after the fourth run. HRP-Cu ^2+^ probably lost its catalytic activity due to the denaturation of HRP in nanoflowers and the deformation of nanoflower shapes of HRP-Cu ^2+^ after each run.

**Table 2:** The polymerization results of guaiacol by free HRP enzyme and H _2_ O _2_ .

Entry ^a^	HRP (mg)	T _p_ (°C)	Yield (%)	T _90_ (°C)	T _50_ (°C)	M _n_ (g/mol)	Ð
12	5	25	16	287	750	6000	1.09
13	5	30	51	296	780	9600	1.13
14	5	40	35	295	756	8000	1.08
15	10	30	53	273	763	10800	1.02
16	15	30	56	289	765	8400	1.04

^a^ All polymerizations were conducted with 100 mg of guaiacol in pH 7.4 PBS buffer in 24 h under in open-air conditions. T _p_ : the polymerization temperature, T _90_ and T _50_ : the temperatures at 90% and 50% residues, respectively. M _n_ : the number average molecular weight, Ð: heterogeneity index.

**Table 3:** Reusability of HRP-Cu ^2+^ in the polymerization of guaiacol.

Entry*	Recycle No	Yield (%)	M _n_ (g/mol)	Ð
17	Run 2	54	14000	1.29
18	Run 3	21	6000	1.10
19	Run 4	8	2900	1.13
20	Run 5	0	-	-

* All polymerizations were carried out with 100 mg of guaiacol, 5 mg of HRP-Cu ^2+^ and H _2_ O _2_ in pH 7.4 PBS buffer at 60 °C under in openair conditions.

## 4. Conclusion

In conclusion, HRP-Cu ^2+^ obtained from the complexation between HRP enzyme and Cu ^2+^ ions exhibited enhanced catalytic activity and stability in the polymerization reaction of guaiacol compared to that of free HRP enzyme. Optimum polymerization of guaiacol was accomplished in pH 7.4 PBS buffer at 60 °C with 5 weight% of HRP-Cu ^2+^ loading in the presence of H _2_ O _2_ and resulted in poly(guaiacol) with 88% yield and 38,000 g/mol molecular weight. Polymerizations using HRP-Cu ^2+^ exhibited poly(guaiacol) with higher yield and molecular weight compared to using the free HRP enzyme. HRP-Cu ^2+^ hybrid nanoflowers also exhibited higher stability at 60 °C and higher reaction temperatures and did not undergo denaturation in contrast with free HRP enzyme. Furthermore, HRP-Cu ^2+^ showed some degree of catalytic activity even after the fourth recycle and can be efficiently used for oxidative polymerizations.
